# Clinical Profile and Comorbidity of Obsessive-Compulsive Disorder among Children and Adolescents: A Cross-Sectional Observation in Bangladesh

**DOI:** 10.1155/2016/9029630

**Published:** 2016-12-14

**Authors:** Md. Hafizur Rahman Chowdhury, Mohammad S. I. Mullick, S. M. Yasir Arafat

**Affiliations:** Department of Psychiatry, Bangabandhu Sheikh Mujib Medical University, Dhaka, Bangladesh

## Abstract

Obsessive compulsive disorder (OCD) is a common disorder characterised by persistent and unwanted intrusive thoughts, images, and urges and repetitive behaviours or mental acts and can cause pervasive impairments. In Bangladesh, the prevalence of OCD among children is 2% which is higher than in previous reporting. This study was aimed at looking into the type, frequency, and severity of symptoms of OCD and comorbidity among children and adolescents. A consecutive 60 OCD cases from a child mental health service with age range of 5–18 years were recruited and divided into below and above 12 years of age group. The assessment was carried out using standardized Bangla version of Development and Wellbeing Assessment and Children's Yale-Brown Obsessive Compulsive Scale was administered. Of the obsession, contamination was the highest followed by doubt, and of the compulsion, washing/cleaning was the highest followed by checking, repeating, and ordering rituals. More than half of the subjects had severe OCD and comorbidity was present in 58% subjects. Specific phobia, social phobia, major depressive disorder, and tic disorder were more prevalent. These symptoms and comorbidity profile can serve the baseline data for a country like Bangladesh and further large scale study would better generalize the study results.

## 1. Introduction

Obsessive-compulsive disorder (OCD) is a relatively common disorder with a lifetime prevalence of 1–3% in children and adolescents, characterised by persistent and unwanted intrusive thoughts, images, and urges (obsessions) and repetitive behaviours or mental acts (compulsions), and typically runs a chronic, wax, and wane course and can cause pervasive impairments in social, academic, and familial functioning [1–6]. Males and females are equally commonly affected from adolescence onwards, but males predominate in prepubertal OCD [7]. The presentation of obsessions and compulsions is heterogeneous in children and adolescents and most of these differences are related to the developmental limitations of younger children compared to adults, and so on [1, 3, 6]. Moreover, OCD is a highly comorbid disorder in childhood with up to 80% of affected children meeting diagnostic criteria for another mental health disorder, most commonly another anxiety disorder, depressive disorder, attention deficit disorder, oppositional defiant disorder (ODD), conduct disorder (CD), or tic disorder, and as many as 50–60% of youth experiencing two or more other mental disorders during their lifetime [3, 6, 8, 9]. A community based epidemiological survey reported that prevalence of OCD among children in Bangladesh is 2% and that is in line with the other cultures [10]. It is evident that the pattern of presentation of obsessive-compulsive disorder between children and adolescent might be different and researchers' clinical observation is that the pattern of presentation of obsessions and compulsions among children and adolescents may be different in Bangladesh due to different cultural and religious background. So, it was aimed to find out the type and frequency of the symptoms of OCD among children and adolescents and to delineate the comorbidity of OCD in children and adolescents in Bangladesh.

## 2. Methods

### 2.1. Ethical Consideration

The researchers were duly concerned about the ethical issues related to the study. Formal ethical clearance was taken from the Institutional Review Board (IRB) of the Bangabandhu Sheikh Mujib Medical University (BSMMU) for conducting the study. Confidentiality of the person and the information was maintained; observed and unauthorized persons did not have any access to the data. Informed written consent was taken from the subjects as well as from the legal guardians after informing the nature and purpose of the study, the procedure of study, the right to refuse, and acceptance and withdrawal from participating in the study and the participants did not gain financial benefit from this study. The present study posed a very low risk to the participants, as procedures such as medical treatments, invasive diagnostics, or procedures causing psychological, spiritual, or social harm were not included.

### 2.2. Design, Subjects, and Instruments

This was a cross-sectional observational study carried out in the Child and Adolescent Outpatient Services of the Department of Psychiatry, BSMMU, Dhaka. Data were collected with the help of convenient sampling during the period of January 2009 to December 2009 from a total of 60 consecutive patients with OCD between 5 and 18 years of age. All children up to 18 years diagnosed as OCD by a psychiatrist were included into the study. Data were collected with the researchers themselves having sound psychopathological background. The cases were divided into two groups: children up to 11 years and adolescents from 12–18 years. For assessment of OCD and comorbidity, structured instrument for assessment of child psychopathology, standardized and validated Bangla parent version of Development and Wellbeing Assessment (DAWABA), was administered [10]. In addition, Self DAWABA was used for the adolescent cases. DAWABA generated ICD-10 DCR [11] diagnoses were assigned for the cases where only Axis one diagnosis was considered. For assessment of symptom pattern and severity of OCD, Children's Yale-Brown Obsessive-Compulsive Scale (CY-BOCS) [12] was used. This scale has five sections: instruction, obsessions checklists, severity items for obsessions, compulsions checklist, and severity items in compulsion. The CY-BOCS comprises 10 severity items, five for the obsessions and five for the compulsions. The severity items assess five aspects pertaining to obsessions and compulsions: frequency, interference, distress, resistance, and control. The 10 severity items are rated on a five-point scale with responses: none, mild, moderate, severe, and extreme for the frequency; interference and distress items are rated as always resists and completely yields to the resistance; and control items are rated as complete control, much control, moderate control, little control, and no control. This scale was translated in Bangla through translation-back translation and committee translation procedure and standardized and validated by the researchers. The translated Bangla version of this scale was used for assessment of pattern and severity of OCD among children and adolescents. Sociodemographic characteristics were assessed by a questionnaire designed for the study. All collected data were cleaned by checking and rechecking for omissions, inconsistencies, and improbabilities. Data were edited, coded, and entered into the computer. After managing data properly, it was analyzed in Statistical Package for Social Science (SPSS) version 12.

## 3. Results

All the 60 respondents were students; 21 were children and 39 were adolescents and 43 (71.7%) were boys and 17 (28.3%) were girls. Their age ranged from 8 to 18 years with the mean of 13.9 ± 3.44 years. Majority of cases came from urban areas (81.7%) with middle income group (57.7%). Forty-five percent of cases had first-degree family history of psychiatric disorder and the highest percentage had OCD (63%) followed by mood disorder (15%) and anxiety disorders (15%).

**Figure 1:**
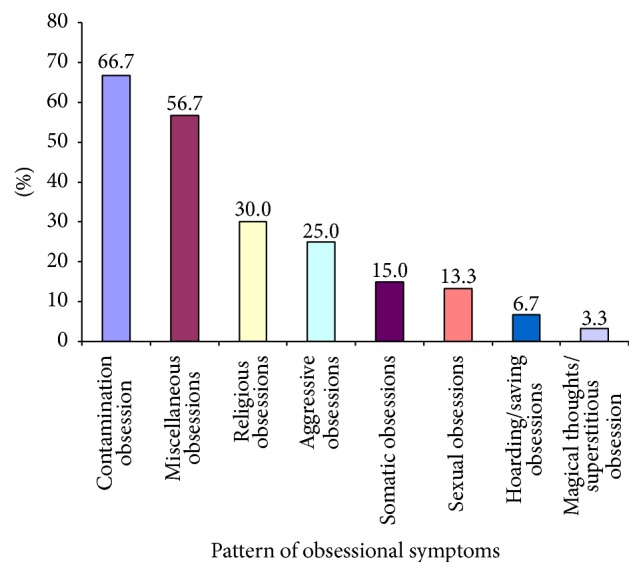
Pattern of obsessive symptoms among 8–18-year-old respondents (*n* = 60).

It was found that the highest percentage of patients had contamination obsession (66.7%) followed by miscellaneous obsessions (56.7%) that mainly included pathological doubt and religious obsession (30%) (Figure 1). Proportion of aggressive obsessions (25.6%), religious obsessions (36%), and miscellaneous obsessions (64%) were found to be higher among the adolescents group whereas contamination obsessions (71%) were found to be high among the children group.

**Figure 2:**
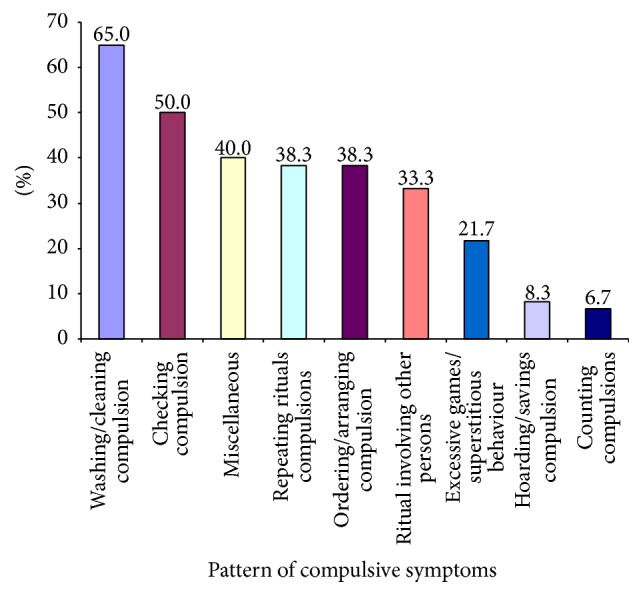
Pattern of compulsive symptoms among 8–18-year-old respondents (*n* = 60).

Regarding compulsive symptoms, the highest percentage of patients had washing/cleaning compulsion (65%) followed by checking compulsion (50%) (Figure 2).

Axis one comorbidity was present among 51.7% of cases with OCD. The highest percentage had specific phobia (10%) followed by major depressive disorder (10%) (Table 1).

Table 2 shows characteristics feature of obsession according to Children's Yale-Brown Obsessive Scale with the mean score of time spent on obsession and obsession-free interval; and interference from obsessions was higher among the adolescents than that of children. Mean obsession score was significantly higher in the adolescents than the children, which was statistically significant and obtained from unpaired Student's *t*-test (Table 2).

Characteristics feature of compulsion according to Children's Yale-Brown Obsessive Scale is shown in Table 3.

Percent distribution of severity of OCD among the respondents according to CY-BOCS revealed that more than half of the patients had severe OCD (53.3%) followed by 36.7% who had extreme OCD, 6.7% who had moderate OCD, and only 3.3% who had mild OCD. The proportion of extreme OCD was found to be high among the adolescents (48.7%) whereas severe OCD was higher among the children (71.4%). The mean score for OCD was 26.81 ± 5.2 for the children and 30.46 ± 5.9 for the adolescents and mean difference was statistically significant (*p* < 0.05).

Table 4 shows relationship between morbidity and selected sociodemographic characteristics of the patients. Analysis found that the OCD with comorbidity was found to be high among the younger patients (61.9%) whereas OCD only was found among the adolescents (53.8%).

## 4. Discussion

This study aimed at looking into the type, frequency, and severity of symptoms of OCD and comorbidity among children and adolescents in Bangladesh and, to the authors' best knowledge, it is the first study to reveal the finding in geographic area as well as the culture.

In the present study, it was found that 45% of patients had 1st-degree family history of psychiatric illness obtained by history. The overall pattern of family history indicated that highest percentage had OCD (63%) followed by mood disorder (15%) and also other anxiety disorders (15%) followed by psychosis (7.4%). However, the proportions of OCD (75%) and mood disorder (16.7%) were found to be high among children whereas, among adolescents, highest percentage had OCD (53.3%) followed by other anxiety disorders. This result gives further evidence that suggests a biological basis for OCD and genetic studies that find higher concordance rates of OCD in first-degree family members and twins than in general population [1–5, 13]. Considering the caregivers' perception about the child's disease, 88.3% believed that it was mental illness, which indicates good awareness among parents of OCD patients, but we should also keep in mind that most of the parents came from good educational and economic background. According to CY-BOCS, the pattern indicates that the highest percentage of patients had contamination obsession (66.7%) followed by miscellaneous obsessions (56.7%) (which mainly includes pathological doubt) and religious obsessions (30%) and these findings are consistent with the findings of other similar types of representative studies [1, 2, 14, 15]. Mean obsession score was significantly higher in the adolescents than the children, which can be explained by cognitive developmental stages. The CY-BOCS pattern of compulsion indicates that the highest percentage of patients had washing/cleaning compulsion followed by checking compulsion and miscellaneous compulsion, and the findings are in agreement with other findings [14, 15]. Present study revealed that the comorbidity was present among 51.7% of children and adolescent OCD patients, which is in between the widely variable range of other studies [3, 5, 6, 13, 16, 17]. However, comorbidity was higher among children (61.9%) than the adolescents (46.2%) but the difference was not statistically significant, reached from Chi square test. This finding (more early onset OCD is associated with more comorbidity) is also consistent with other studies' findings [18]. The pattern of comorbidity also indicated that the highest percentage had specific phobia followed by major depressive disorder, social phobia, tic disorder, oppositional defiant disorder, hyperkinetic disorder, generalized anxiety disorder, conduct disorder, mental retardation, and autism spectrum disorder. This finding is partly comparable to other findings except absence of alcohol and drug related disorders and eating disorder, which can be explained by Bangladeshi sociocultural background [19, 20]. Comorbidity pattern also showed two specific patterns for children and adolescent groups. The proportions of major depressive disorder (15.4%) followed by social phobia (12.8%), generalized anxiety disorder (5.1%), and conduct disorder (5.1%) were higher among the adolescents whereas specific phobia (28.6%), tic disorder (23.8%), hyperkinetic disorder (19%), mental retardation (4.8%), and autism spectrum disorder were higher among the children. This finding is somewhat consistent with other studies' findings [3, 5, 6, 13, 21].

## 5. Conclusion

Though generalization of the study finding may be difficult because of small sample size, single centre exposer, and tertiary care level coverage, it is the first study to explore the issue; and findings of the study will help our health professionals to address the symptom pattern of OCD, its severity, and comorbid conditions among child and adolescent patients in Bangladesh. It will increase the space and will help creating an information baseline to carry out further study in this field in future. Further, large-scale, multicentre study can be conducted to explore OCD symptoms with severity adequately and necessary policies can be formulated based on the study result.

## Figures and Tables

**Table 1:** Pattern of comorbidity among 8–18-year-old respondents.

Pattern of comorbidity	Age in years				Total (*n* = 60)	
	<12 (*n* = 21)		≥12 (*n* = 39)			
	Number	%	Number	%	Number	%
Generalized anxiety disorder	0	.0	2	5.1	2	3.3
Specific phobia	6	28.6	0	.0	6	10.0
Tic disorder	5	23.8	0	.0	5	8.3
Conduct disorder	0	.0	2	5.1	2	3.3
Oppositional defiant disorder	2	9.5	2	5.1	4	6.7
Hyperkinetic disorder	4	23.8	0	.0	4	9.0
Major depressive disorder	0	.0	6	15.4	6	10.0
Social phobia	1	4.8	5	12.8	6	10.0
Autism spectrum disorder	1	4.8	1	2.6	2	3.3
Comorbidity						
OCD only	8	38.1	21	53.8	29	48.3
Comorbidity	13	61.9	18	46.2	31	51.7

**Table 2:** Characteristics feature of obsession according to Children's Yale-Brown Obsessive Scale.

Characteristics	Age in years				Total (*n* = 60)		*p* value
	<12 (*n* = 21)		≥12 (*n* = 39)				
	Number	%	Number	%	Number	%	
Time spent on obsession							
Mild	2	9.5	2	5.1	4	6.7	
Moderate	9	42.9	10	25.6	19	31.7	
Severe	10	47.6	15	38.5	25	41.7	
Extreme	0	.0	12	30.8	12	20.0	
Mean ± SD score	2.38 ± 0.7		2.95 ± 0.9		2.75 ± 0.9		0.013

Obsession-free interval							
Long	1	4.8	3	7.7	4	6.7	
Moderately long	16	76.2	14	35.9	30	50.0	
Short	3	14.3	17	43.6	20	33.3	
Extremely short	1	4.8	5	12.8	6	10.0	
Mean ± SD score	2.19 ± 0.6		2.62 ± 0.8		2.47 ± 0.8		0.040

Interference from obsessions							
Long	1	4.8	3	7.7	4	6.7	
Moderately long	10	47.6	7	17.9	17	28.3	
Short	10	47.6	10	25.6	20	33.3	
Extremely short	0	.0	19	48.7	19	31.7	
Mean ± SD score	2.43 ± 0.6		3.15 ± 1.0		2.90 ± 0.9		0.003

Distress of obsessions							
Long	1	4.8	2	5.1	3	5.0	
Moderately long	4	19.0	3	7.7	7	11.7	
Short	12	57.1	12	30.8	24	40.0	
Extremely short	4	19.0	22	56.4	26	43.3	
Mean ± SD score	2.90 ± 0.8		3.38 ± 0.8		3.22 ± 0.8		0.035

Resistance							
Much resistance	1	4.8	1	2.6	2	3.3	
Some resistance	1	4.8	3	7.7	4	6.7	
Often yields	17	81.0	23	59.0	40	66.7	
Completely yields	2	9.5	12	30.8	14	23.3	
Mean ± SD score	2.95 ± 0.6		3.18 ± 0.7		3.10 ± 0.7		0.204

Control over obsessions							
Much control	1	4.8	0	.0	1	1.7	
Moderate control	1	4.8	3	7.7	4	6.7	
Little control	17	81.0	23	59.0	40	66.7	
No control	2	9.5	13	33.3	15	25.0	
Mean ± SD score	2.95 ± 0.6		3.26 ± 0.6		3.15 ± 0.6		0.063
Total obsession score	13.62 ± 2.8		15.92 ± 3.1		15.12 ± 3.2		0.006

*p* value reached from unpaired Student's *t*-test.

**Table 3:** Characteristics feature of compulsion according to Children's Yale-Brown Compulsive Scale.

Characteristics	Age in years				Total (*n* = 60)		*p* value
	<12 (*n* = 21)		≥12 (*n* = 39)				
	Number	%	Number	%	Number	%	
Time spent on compulsion							
Mild	1	4.8	2	5.1	3	5.0	
Moderate	9	42.9	16	41.0	25	41.7	
Severe	11	52.4	13	33.3	24	40.0	
Extreme	0	.0	8	20.5	8	13.3	
Mean ± SD score	2.48 ± 0.6		2.69 ± 0.9		2.62 ± 0.8		0.312
Compulsion-free interval							
Long	1	4.8	2	5.1	3	5.0	
Moderately long	15	71.4	21	53.8	36	60.0	
Short	5	23.8	13	33.3	18	30.0	
Extremely short	0	.0	3	7.7	3	5.0	
Mean ± SD score	2.19 ± 0.5		2.44 ± 0.7		2.35 ± 0.7		0.171
Interference from compulsions							
Long	1	4.8	2	5.1	3	5.0	
Moderately long	11	52.4	12	30.8	23	38.3	
Short	9	42.9	19	48.7	28	46.7	
Extremely short	0	.0	6	15.4	6	10.0	
Mean ± SD score	2.38 ± 0.6		2.74 ± 0.8		2.62 ± 0.7		0.069
Distress of compulsions							
Long	1	4.8	2	5.1	3	5.0	
Moderately long	5	23.8	6	15.4	11	18.3	
Short	13	61.9	15	38.5	28	46.7	
Extremely short	2	9.5	16	41.0	18	30.0	
Mean ± SD score	2.76 ± 0.7		3.15 ± 0.9		3.02 ± 0.8		0.082
Resistance							
1	1	4.8	1	2.6	2	3.3	
2	4	19.0	8	20.5	12	20.0	
3	14	66.7	22	56.4	36	60.0	
Completely yields	2	9.5	8	20.5	10	16.7	
Mean ± SD score	2.81 ± 0.7		2.95 ± 0.7		2.90 ± 0.7		0.471
Control over compulsion							
Much control	1	4.8	1	2.6	2	3.3	
Moderate control	5	23.8	8	20.5	13	21.7	
Little control	13	61.9	20	51.3	33	55.0	
No control	2	9.5	10	25.6	12	20.0	
Mean ± SD score	2.76 ± 0.7		3.00 ± 0.8		2.92 ± 0.7		0.240
Total obsession score	13.19 ± 2.8		14.54 ± 3.4		14.07 ± 3.2		0.121

*p* value reached from unpaired Student's *t*-test.

**Table 4:** Relationship between morbidity and selected sociodemographic characteristics.

Characteristics	Number	Morbidity				*p* value
		OCD only		Comorbidity		
		Number	%	Number	%	
Age in years						
<12	21	8	38.1	13	61.9	0.244
≥12	39	21	53.8	18	46.2	
Sex						
Male	43	24	55.8	19	44.2	0.065
Female	17	5	29.4	12	70.6	
Residence						
Urban	49	25	51.0	24	49.0	0.379
Rural	11	4	36.4	7	63.6	
Religion						
Muslim	54	25	46.3	29	53.7	0.344
Non-Muslim	6	4	66.7	2	33.3	
Level of education						
Primary	17	6	35.3	11	64.7	0.204
Secondary and above	43	23	53.5	20	46.5	
Father's level of education						
Below graduate	19	10	52.6	9	47.4	0.650
Graduate and above	41	19	46.3	22	53.7	
Monthly family income (Tk.)						
Up to US $280	31	15	48.4	16	51.6	0.993
> US $280	29	14	48.3	15	51.7	

*p* value reached from Chi square test.

## Abbreviations

OCD: Obsessive-compulsive disorder
IRB: Institutional Review Board
BSMMU: Bangabandhu Sheikh Mujib Medical University
DAWBA: Development and Wellbeing Assessment
DCR: Diagnostic criteria for research
CY-BOCS: Children's Yale-Brown Obsessive-Compulsive Scale
SPSS: Statistical Package for Social Science.
